# Pre-procedural abnormal function of von Willebrand Factor is predictive of bleeding after surgical but not transcatheter aortic valve replacement

**DOI:** 10.1007/s11239-019-01917-7

**Published:** 2019-07-29

**Authors:** Kajetan Grodecki, Karol Zbroński, Elżbieta Przybyszewska-Kazulak, Anna Olasińska-Wiśniewska, Radosław Wilimski, Bartosz Rymuza, Piotr Scisło, Paweł Czub, Dominika Koper, Janusz Kochman, Katarzyna Pawlak, Olga Ciepiela, Marek Grygier, Marek Jemielity, Maciej Lesiak, Krzysztof J. Filipiak, Grzegorz Opolski, Zenon Huczek

**Affiliations:** 1 1st Department of Cardiology, Medical University of Warsaw, 1a Banacha, 02-097 Warsaw, Poland; 2 Department of Laboratory Diagnostics, Medical University of Warsaw, Warsaw, Poland; 3 1st Department of Cardiology, Poznan University of Medical Sciences, Poznan, Poland; 4 Department of Cardiac Surgery, Medical University of Warsaw, Warsaw, Poland; 5 Department of Cardiac Surgery and Transplantology, Poznan University of Medical Sciences, Poznan, Poland

**Keywords:** von Willebrand Factor, Aortic stenosis, Transcatheter aortic valve implantation, Surgical aortic valve implantation, Bleeding complications

## Abstract

Both transcatheter aortic valve implantation (TAVI) and surgical aortic valve replacement (SAVR) have been proven to effectively correct von Willebrand Factor (vWF) pathologies, however there is limited data simultaneously comparing outcomes of both approaches. We prospectively enrolled patients with severe aortic stenosis referred for TAVI (n = 52) or SAVR (n = 48). In each case, vWF antigen (vWF:Ag), vWF activity (vWF:Ac) and activity-to-antigen (vWF:Ac/Ag) ratio were assessed at baseline, 24 h and 72 h after procedure. VWF abnormalities were defined as reduced vWF:Ac/Ag ratio (< 0.8). Bleeding events in both arms were classified according to Valve Academic Research Consortium (VARC-2) definitions. Overall, there was no difference between patients referred for TAVI and SAVR in vWF:Ac (1.62 ± 0.52 vs 1.71 ± 0.64; p = 0.593), vWF:Ag (1.99 ± 0.81 vs 2.04 ± 0.81; p = 0.942) or vWF:Ac/Ag ratio (0.84 ± 0.16 vs 0.85 ± 0.12; p = 0.950). Pathological vWF:Ac/Ag ratio was found in 20 (38%) TAVI and 15 (31%) SAVR patients (p = 0.407). Normalization of vWF:Ac/Ag ratio at day 3 after procedure was achieved in 19 (95%) TAVI and 13 (87%) SAVR patients (p = 0.439). Similar prevalence of major or life-threatening bleedings (MLTB) after TAVI and SAVR in entire groups was observed (19% vs. 23%, p = 0.652). VWF abnormalities were associated with higher incidence of MLTB in SAVR (53% vs 9%, p < 0.001), but not TAVI (15% vs. 22%, p = 0.132). Accordingly, in receiver-operating characteristic curve analysis vWF:Ac/Ag ratio < 0.8 showed significant sensitivity and specificity for predicting MLTB in SAVR group (AUC 0.735, 95% CI 0.538–0.931, p = 0.019). We proved that abnormal function of vWF is corrected successfully after both TAVI and SAVR, but vWF abnormalities are predictive of MLTB only in surgical patients.

**Electronic supplementary material:**

The online version of this article (10.1007/s11239-019-01917-7) contains supplementary material, which is available to authorized users.

## Highlights

- Von Willebrand Factor abnormalities are associated with severity of aortic stenosis.
- Both TAVI and SAVR are effective treatment for vWF abnormalities.
- Baseline vWF abnormalities predict MLTB only in surgical patients.
- Von Willebrand Factor studies should be continued to improve stratification of surgical patients at risk of postoperative bleeding.

## Introduction

Aortic stenosis (AS) leads inevitably to structural and functional changes in heart muscle, but affects also the blood components. The high-shear hemodynamics of stenotic aortic valve disrupts erythrocyte membranes and decreases platelet function [1]. Moreover, a large multimeric molecule of von Willebrand Factor (vWF) has been shown to undergo conformational changes as the high-velocity jet passes through the narrowed orifice [2].

VWF is the protein that plays key-role in primary hemostasis by mediating adhesion of platelets to the sites of vascular damage and shows high pro-haemostatic properties as high-molecular weight (HMW) multimer [3]. In the form of globularly shaped HMW multimers, vWF remains sensitive to shear stress, which leads to its unfolding and elongation into long-chain molecule [4]. Mechanism of conformational changes enhances functional properties of macromolecule, but is associated with exposure of binding sites for metalloprotease ADAMTS-13 that cleaves HMW multimers into smaller and less active molecules [5]. While enzymatic proteolysis counterregulates prothrombotic function of unfolded vWF strings, increased loss of HMW vWF multimers in AS leads to acquired von Willebrand syndrome (avWS) [6].

Initially linked with gastrointestinal bleeding from angiodysplasia (Heyde syndrome), vWF abnormalities are likely to underlie wider range of bleeding disorders accompanying AS. Previous studies showed that significant reduction of HMW vWF multimers is present in 20% to 70% patients with moderate to severe AS and gastrointestinal bleeding in the setting of vWF deficiency has been reported in up to 20% [7, 8]. Both transcatheter aortic valve implantation (TAVI) and surgical aortic valve replacement (SAVR) have been proven to effectively correct vWF pathologies [9–12].

Additionally, vWF emerged recently as novel biomarker useful in management of patients with valvular heart diseases [13]. However, most of the studies focus on transcatheter therapies and little present-day data is available on this topic in the context of SAVR. We therefore sought to compare effect of surgical and transcatheter approaches in treatment of vWF abnormalities. Furthermore, we evaluated the usefulness of a novel latex-based immunoturbidimetric assay for detection of vWF activity to predict bleeding complications following TAVI and SAVR.

## Materials and methods

### Study design and population

Patients with severe symptomatic aortic stenosis who were referred for TAVI or SAVR at two participating centers (First Department of Cardiology, Medical University of Warsaw, Warsaw, Poland and First Department of Cardiology, University of Medical Sciences, Poznan, Poland) were prospectively enrolled in the study between April 2015 and January 2017.

Severe aortic stenosis was defined as aortic valve area < 1.0 cm^2^; or indexed valve area < 0.6 cm^2^/m^2^; or mean gradient > 40 mmHg; or maximum jet velocity > 4.0 m/s; or velocity ratio < 0.25 [14].

The decision on patient qualification for TAVI or SAVR was made by the local Heart Team after careful evaluation of each case.

In TAVI: mechanically-, and self-expandable aortic valve prostheses of the second generation were used, transfemoral access with two ProGlides (Abbott Vascular, Abbott Park, Illinois, USA) for vascular closure was applied in all cases, procedures were performed in hybrid operating rooms under general anesthesia or local anesthesia with conscious sedation. Transcathether aortic valve-in-valve implantation was excluded from the study.

In SAVR: stented and stentless aortic valve bioprostheses were used and minimally invasive aortic valve replacement approach was applied in all cases. Exclusion criteria included procedures such as coronary artery bypass grafting, mitral or tricuspid valve surgery, or aortic surgery combined with SAVR or application of mechanical aortic valve prosthesis.

Informed consent was obtained from all participating patients and local ethics committee granted permission for the study.

### Periprocedural antithrombotic regimens

#### TAVI

Regarding periprocedural pharmacological treatment, patients taking no anticoagulation, where given loading doses of 300 mg of aspirin and clopidogrel within 24 h before TAVI, and then continued with 75 mg daily after the procedure.

Oral antiplatelet drugs were continued throughout the hospitalization, unless major bleeding occurred. Patients on chronic oral anticoagulant (OAC) treatment, where switched to low-molecular weight heparin (LMWH) at least 48 h before the start of the procedure. After TAVI they were treated with double therapy (either aspirin + OAC in patients without recent stent implantation or clopidogrel + OAC in patients with recent PCI).

During the procedure, unfractionated heparin (UFH) was administered to maintain an activated clotting time (ACT) > 300 s with subsequent reversal using protamine sulfate (1 g/100 units of heparin given).

#### SAVR

Patients on oral anticoagulation before surgery were switched to low-molecular weight heparin (LMWH) at least 48 h before SAVR.

During the procedure, UFH was administered prior to cardiopulmonary bypass (CPB) initiation to maintain an activated clotting time ACT > 400 s with subsequent reversal using protamine sulfate following weaning from CPB (1 g/100 units of heparin given).

In patients with aortic bioprosthetic valves who were in sinus rhythm after the operation and had no other indication for oral anticoagulation, LMWH was administered during hospital stay and aspirin 75–100 mg/day was started on the first postoperative day and continued for 3 months. In patient with aortic bioprosthetic valve and indication for oral anticoagulation (most commonly atrial fibrillation) LMWH was continued until anticoagulation with a vitamin K antagonist (VKA) achieved an international normalized ration (INR) of 2.5.

### Blood sampling and laboratory assays

For all the study participants, venous peripheral blood samples were collected at three time-points: day before procedure, as well as on the first and third day after procedure. Blood samples were taken into standardized blood collection tubes containing 3.2% trisodium citrate in the room temperature and were centrifuged within 30 min accordingly to manufacturer’s instruction (15 min at 1500×*g*). Obtained plasma was subsequently frozen at − 80 °C and stored for no longer than 6 months.

von Willebrand Factor was characterized with: (1) vWF activity (vWF:Ac; INNOVANCE® VWF Ac); (2) vWF antigen (vWF:Ag; VWF Ag®); and activity-to-antigen (WF:Ac/vWF:Ag) ratio. Standard human plasma was used for calibration (all Siemens Healthcare Diagnostics, Eschborn, Germany). Measurements were performed within the routine laboratory analysis using Siemens Behring Coagulation System XP®.

### Endpoint definitions

VWF abnormalities were defined as reduced vWF activity (< 0.46 or < 0.61 IU/mL depending on blood type) and/or reduced vWF activity-to-antigen ratio (< 0.8) [15]. Possible avWS was defined as the presence of the history of bleeding symptoms plus vWF abnormalities. Clinically suspected Heyde’s syndrome was defined as the presence of severe aortic stenosis alongside a history of gastrointestinal angiodysplasia and gastrointestinal bleeding documented by endoscopy [11].

To evaluate bleeding complications Valve Academic Research Consortium-2 (VARC-2) definitions were applied. Bleeding events were classified as: life-threatening, major and minor [16].

### Sample size calculation

The standard deviation (SD) and mean difference between the two treatment arms were estimated based on previous studies utilizing functional vWF assays in patients treated with TAVI and SAVR [10, 11]. The required sample size was calculated by a two-sided *t* test at a significance level of 0.05 with the following assumptions: (1) SD in each group ± 0.2; (2) mean difference between the groups = 0.12; (3) nominal test power = 0.8. Based on this sample size estimation, a total of 90 patients (45 per group) should be enrolled in the trial.

### Statistical analysis

Continuous variables are expressed as the mean ± standard deviation were compared with Student’s *t* test, Mann–Whitney U test or Wilcoxon signed-rank test as appropriate. *χ*^2^ or Fisher’s exact tests were used for comparison of categorical variables expressed as counts and percentages. The correlation between two continuous variables was measured with the bivariate Pearson correlation. In order to assess discrimination ability of vWF parameters in terms of predicting major and life-threatening bleeding, receiver-operating characteristic (ROC) analysis with an area under the curve (AUC [c-statistic]) was performed. All probability values reported are two-sided and a value < 0.05 was considered to be significant. All data were processed using SPSS software, version 23 (IBM SPSS Statistics, New York, US).

## Results

### Patients and baseline characteristics

The study population included 52 patients treated with TAVI and 48 patients treated with SAVR. At baseline, vWF abnormalities were found in 20 (38%) TAVI and 15 (31%) SAVR patients, (p = 0.407). Possible avWS was diagnosed in 3 (15%) transcatheter and 3 (20%) surgical patients (p = 0.698). One surgical patient had clinically suspected Heyde’s syndrome.

No differences in demographic data and comorbidities were found between patients with and without vWF abnormalities neither in TAVI nor SAVR group (detailed data is presented in Table 1). Subjects with vWF abnormalities had similar rates of preoperative bleedings compared to patients without such abnormalities in both: TAVI (15% vs 22%, p = 0.540) and SAVR (20% vs 18%, p = 0.881) populations. Baseline echocardiographic examination did not show any significant disparities between patients with and without vWF abnormalities, except for the values of aortic valve area in transcatheter (0.61 ± 0.13 vs 0.76 ± 0.19, p = 0.012), as well as surgical (0.69 ± 0.17 vs 0.79 ± 0.24, p = 0.043) cohort.

**Table 1:** Demographics, comorbidities, and echocardiographic data of cohorts with and without von Willebrand Factor abnormalities

	TAVI (n = 52)			SAVR (n = 48)		
	vWF abnormalities (n = 20)	no vWF abnormalities (n = 32)	p value	vWF abnormalities (n = 15)	no vWF abnormalities (n = 33)	p value
Demographic data						
Mean age (years)	77.8 ± 5.1	77.7 ± 6.7	0.528	63.4 ± 9.4	66.1 ± 11.5	0.261
Male sex	12 (60)	17 (53)	0.776	10 (67)	21 (64)	1.000
Logistic EuroSCORE (%)	20.7 ± 13.6	18.67 ± 12.36	0.792	5.69 ± 6.11	4.54 ± 4.28	0.252
STS-PROM (%)	4.94 ± 2.84	4.92 ± 3.36	0.735	1.04 ± 0.73	1.48 ± 1.24	0.459
Diabetes mellitus	9 (45)	15 (47)	1.000	3 (20)	10 (30)	0.727
Arterial hypertension	15 (75)	24 (75)	1.000	9 (60)	21 (64)	1.000
Dyslipidaemia	15 (75)	26 (81)	0.730	11 (73)	20 (61)	0.521
COPD	4 (20)	6 (19)	1.000	0 (0)	4 (12)	0.294
Atrial fibrillation	9 (45)	9 (28)	0.244	2 (13)	9 (27)	0.462
CKD	8 (40)	19 (59)	0.255	3 (20)	10 (30)	0.727
History of stroke/TIA	1 (5)	3 (9)	1.000	2 (13)	0 (0)	0.093
CAD	10 (50)	16 (50)	1.000	5 (33)	12 (36)	1.000
History of any bleeding	3 (15)	7 (22)	0.540	3 (20)	6 (18)	0.881
History of GI bleeding	2 (10)	3 (9)	0.940	2 (13)	3 (9)	0.655
Antithrombotic treatment						
Antiplatelets						
SAPT	8 (40)	15 (47)	0.776	8 (53)	10 (30)	0.198
DAPT	4 (20)	7 (22)	1.000	1 (7)	3 (9)	1.000
Anticoagulants	9 (45)	9 (28)	0.244	1 (7)	10 (30)	0.136
Preprocedural TTE						
LVEF (%)	47.3 ± 15.3	53.8 ± 14.6	0.416	61.2 ± 8.6	55.9 ± 10.9	0.111
AVA (cm^2^)	0.61 ± 0.13	0.76 ± 0.19	0.012	0.69 ± 0.17	0.79 ± 0.24	0.043
Vmax (m/s)	4.4 ± 0.4	4.2 ± 0.9	0.181	4.4 ± 0.7	4.4 ± 0.9	0.772
Mean PG (mmHg)	52.3 ± 14.1	41.7 ± 18.5	0.062	51.2 ± 14.9	49.4 ± 19.3	0.306
Max PG (mmHg)	84.0 ± 19.8	74.5 ± 32.0	0.284	83.8 ± 22.0	82.4 ± 30.4	0.456
Predischarge TTE						
LVEF (%)	53.6 ± 11.2	54.9 ± 12.0	0.582	61.0 ± 7.8	55.8 ± 10.8	0.089
AVA (cm^2^)	1.72 ± 0.56	1.84 ± 0.61	0.305	1.92 ± 0.28	2.04 ± 0.39	0.620
Vmax (m/s)	1.8 ± 0.6	1.9 ± 0.4	0.183	2.4 ± 0.4	2.4 ± 0.4	0.650
Mean PG (mmHg)	9.1 ± 4.2	8.5 ± 3.9	0.409	16.4 ± 7.7	14.4 ± 6.0	0.350
Max PG (mmHg)	16.6 ± 6.9	16.7 ± 6.6	0.951	29.9 ± 12.8	26.4 ± 8.9	0.476
AR of at least moderate degree	1 (5)	2 (7)	1.000	0 (0)	0 (0)	n/a

Values are n (%) or mean ± SD

*AR* aortic regurgitation, *AVA* aortic valve area, *AVAi* aortic valve area indexed for body surface, *CAD* coronary artery disease, *CKD* chronic kidney disease, *COPD* chronic obstructive pulmonary disease, *DAPT* dual antiplatelet therapy, *LVEF* left ventricular ejection fraction, *PG* pressure gradient, *SAPT* single antiplatelet therapy, *TTE* transthoracic echo, *TIA* transient ischemic attack, *Vmax* peak velocity

In TAVI cohort: 43 patients received a self-expandable CoreValve Evolut R bioprosthesis, and 9 patients received a mechanically expandable Boston Scientific Lotus Valve. In SAVR cohort: 40 patients received a stented bioprostheses (30 Labcor Dokimos Plus, 4 St Jude Medical Epic, 6 Edwards Magna Ease 3000) and 8 patients received stentless Sorin Freedom Solo bioprostheses.

### Baseline vWF parameters

Overall, there was no difference between patients referred for TAVI and SAVR in vWF:Ac (1.62 ± 0.52 vs 1.71 ± 0.64; p = 0.593), vWF:Ag (1.99 ± 0.81 vs 2.04 ± 0.81; p = 0.942) or vWF:Ac/Ag ratio (0.84 ± 0.16 vs 0.85 ± 0.12; p = 0.950).

**Fig. 1:**
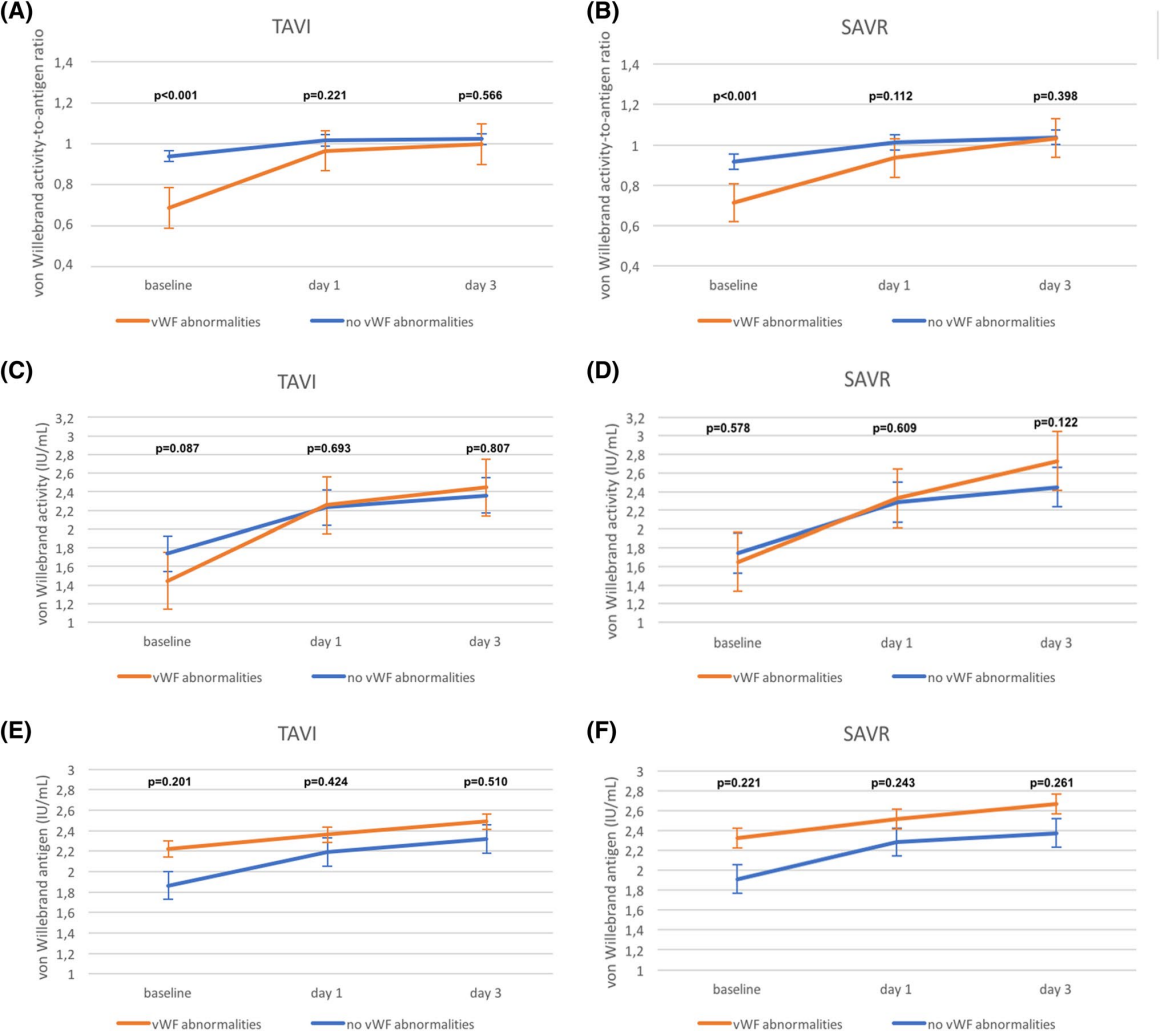
Comparison of von Willebrand Factor parameters according to the treatment strategy: **a** von Willebrand activity-to-antigen ratio in TAVI patients; **b** von Willebrand activity-to-antigen ratio in SAVR patients; **c** von Willebrand activity in TAVI patients; **d** von Willebrand activity in SAVR patients; **e** von Willebrand antigen in TAVI patients; **f** von Willebrand antigen in SAVR patients

In transcatheter as well surgical cohorts, the vWF:Ac/vWF:Ag ratio was lower in patients with vWF abnormalities compared to patients without vWF abnromalities (0.68 ± 0.10 vs 0.94 ± 0.10, p < 0.001 for TAVI; 0.71 ± 0.05 vs 0.92 ± 0.10, p < 0.001 for SAVR; Fig. 1a, b).

In TAVI population no differences between patients with and without vWF abnormalities were found neither in mean values of vWF:Ac (1.44 ± 0.37 vs 1.74 ± 0.58, p = 0.087; Fig. 1c) nor mean vWF:Ag (2.21 ± 1.01 vs 1.86 ± 0.63, p = 0.201; Fig. 1e). Similar results were noted for SAVR patients with and without vWF abnormalities, in whom both vWF:Ac (1.64 ± 0.68 vs 1.74 ± 0.63, p = 0.578; Fig. 1d) and vWF:Ag (2.32 ± 0.96 vs 1.91 ± 0.71, p = 0.221; Fig. 1f) were not significantly different.

Bivariate Pearson correlation was performed for baseline markers of vWF function (vWF:Ac, vWF:Ag and WF:Ac/vWF:Ag ratio) with different markers of aortic-valve-stenosis severity (aortic valve area, peak velocity, mean and max pressure gradients). Mean pressure gradient showed significant negative correlation with both vWF:Ac (r = − 0.226, p = 0.024; Supplementary Fig. 1a) and vWF:Ac/vWF:Ag ratio (r = − 0.240, p = 0.016; Supplementary Fig. 1b). Moreover, peak velocity negatively correlated with vWF:Ac (r = − 0.216, p = 0.031 Supplementary Fig. 1c).

### Postprocedural evolution of vWF parameters

Normalization of vWF:Ac/Ag ratio at day 3 after the procedure was achieved in 19 (95%) TAVI and 13 (87%) SAVR patients (p = 0.439). Disparities between patients with and without vWF abnormalities observed in baseline vWF parameters were resolved from the first post-interventional day. In TAVI patients with baseline vWF abnormalities, vWF:Ac/Ag ratio increased to 0.96 ± 0.11, while in group without abnormalities increase to 1.01 ± 0.12 was noted on the first day (p = 0.221; Fig. 1a). On the third day, vWF:Ac/Ag ratio values were: 0.99 ± 0.14 and 1.02 ± 0.08, respectively (p = 0.566). Similarly, in surgical patients with baseline vWF abnormalities, vWF:Ac/Ag ratio increased to 0.94 ± 0.15 and in subjects without vWF abnormalities increase to 1.01 ± 0.12 was observed on the first postoperative day (p = 0.112; Fig. 1b). In SAVR group, on the third day, vWF:Ac/Ag ratio was 1.03 ± 0.15 and 1.04 ± 0.09, respectively (p = 0.398). Evolution of remaining parameters in transcatheter and surgical groups is presented in Fig. 1c–e.

**Fig. 2:**
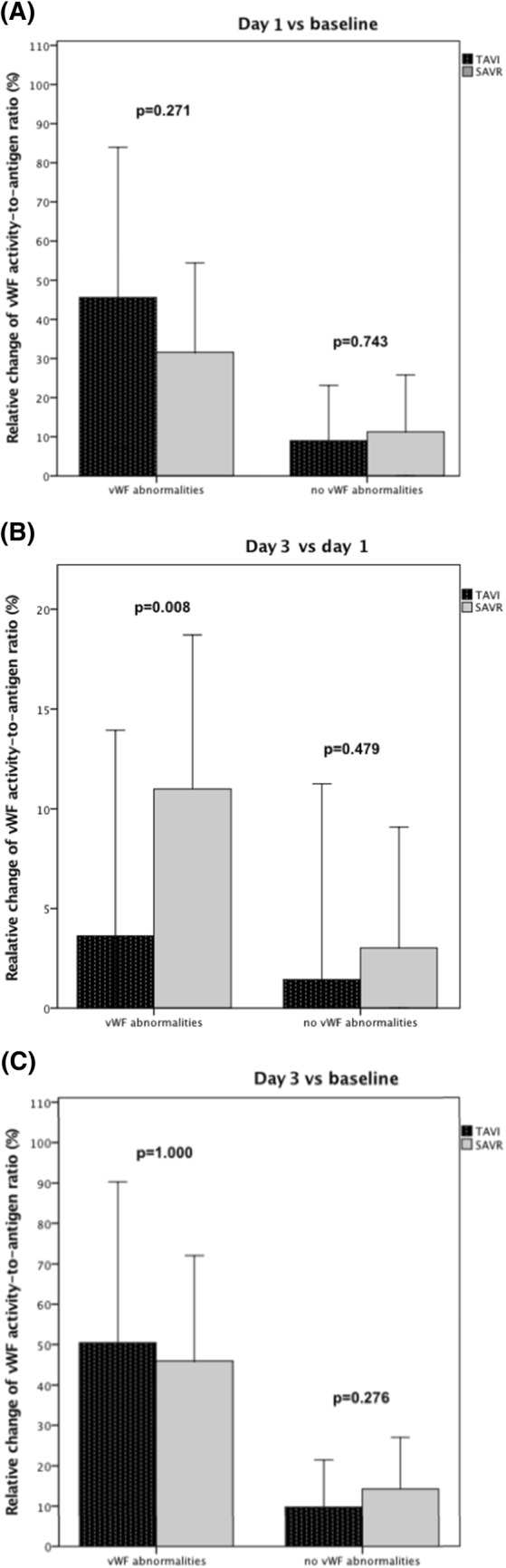
Relative change of von Willebrand Factor activity-to-antigen ratio: **a** on day 1 in comparison to baseline; **b** on day 3 in comparison to day 1; **c** on day 3 in comparison to baseline

No differences in relative increase of vWF:Ac/Ag ratio were found on the first post-interventional day between TAVI and SAVR neither in patients with (46 ± 38% vs 32 ± 22%, p = 0.271; Fig. 2a) nor without (9 ± 14% vs 11 ± 14%, p = 0.743) vWF abnormalities. Patients with vWF abnormalities in transcatheter population, however, were characterized with lower increase on the third post-interventional day when compared to surgical group (4 ± 10% vs 11 ± 7%, p = 0.008 vs day 1; Fig. 2b). In patients without baseline vWF abnormalities such a disparity was not observed (2 ± 10% vs 3 ± 6%, p = 0.479 vs day 1; Fig. 2b). No differences were found between transcatheter and surgical approach in overall relative increase of vWF:Ac/Ag ratio neither in patients with (50 ± 40% vs 46 ± 26%, p = 1.000; Fig. 2c) nor without vWF abnormalities (10 ± 12% vs 14 ± 13%, p = 0.276; Fig. 2c). Both TAVI and SAVR had comparable effect on vWF:Ac improvement in patients with and without vWF abnormalities (Supplementary Fig. 2a–c).

Size of bioprosthesis was not found to impact evolution of vWF parameters neither in transcatheter nor surgical patients (Supplementary Material 1).

### Bleeding complications and mortality

**Fig. 3:**
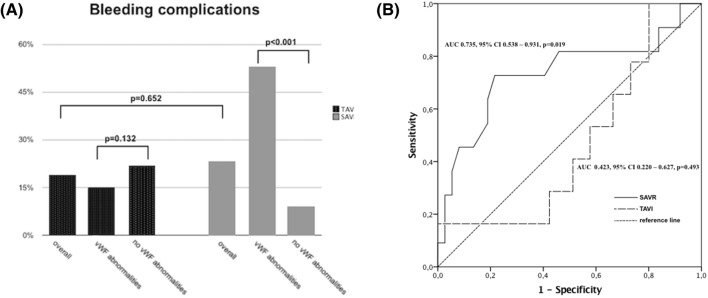
**a** Bleeding complication in TAVI and SAVR cohorts. **b** Receiver operating characteristics (ROC) curves showing sensitivity and specify of von Willebrand Factor activity-to-antigen ratio for prediction of major or life-threatening bleedings in TAVI and SAVR cohorts

Overall, the frequency of bleeding complications was comparable between TAVI and SAVR (19% vs. 23%, p = 0.652; Fig. 3a). Specific causes of major and life-threatening bleeding complication stratified by von Willebrand abnormalities in both arms are shown in Table 2.

**Table 2:** Causes of periprocedural major or life-threatening bleeding complications stratified by presence of von Willebrand Factor abnormalities in TAVI and SAVR groups

	vWF abnormalities	No vWF abnormalities
TAVI	(n = 20)	(n = 32)
Ilio-femoral injury	1 (5)	2 (6)
Pseudo-aneursym	0 (0)	1 (3)
Access-site bleeding incl. large groin hematoma	2 (10)	4 (13)

SAVR	(n = 15)	(n = 33)
Tamponade	1 (6)	1 (3)
Hemothorax	1 (6)	0 (0)
Increased chest output	4 (27)	2 (6)
Chest wall/sternum-related bleeding	2 (13)	0 (0)

In TAVI cohort, no difference was found between subjects with and without von Willebrand abnormalities in terms of major or life-threatening bleeding (15% vs 22%, p = 0.132; Fig. 3a). ROC curve analysis was performed for baseline markers of vWF function to assess their ability to predict major or life-threatening bleeding complications in transcatheter patients, but none showed significant sensitivity and specificity (AUC for vWF:Ac/vWF:Ag ratio was 0.423, 95% CI 0.220–0.627, p = 0.493; Fig. 3b).

In SAVR cohort, patients with von Willebrand abnormalities suffered more frequently from major or life-threatening bleeding (53% vs 9%, p < 0.001; Fig. 3a) than the remaining. ROC curve analysis of baseline vWF function parameters showed good sensitivity and specificity of the vWF:Ac/vWF:Ag ratio (AUC 0.735, 95% CI 0.538–0.931, p = 0.019; Fig. 3b) in predicting MLTB of surgical patients.

Additionally, preprocedural presence of vWF abnormalities did not increase 1-year mortality in neither transcatheter (10% vs. 12.5%, p = 0.783) nor surgical cohort (6.5% vs. 3%, p = 0.559).

## Discussion

The main finding of the present study is that that surgical and transcatheter strategies have similar effect on improvement of vWF parameters. To the best of our knowledge, this is the first direct comparison of two approaches. Moreover, it was found that vWF abnormalities are predictive of MLTB in surgical patients.

Prevalence of abnormal vWF multimers in AS patients varies considerably across studies—ranging mostly between 20 and 70% [7, 8]. Differences arise from inconsistent methodology of screening for vWF abnormalities as there are multiple assays available: vWF-ristocetin cofactor activity (vWF:RCo), vWF collagen binding (vWF:CB), closure time of a membrane coated with collagen and adenosine-5′-diphosphate (CT-ADP) with Platelet Function Analyzer (PFA) and finally—considered to be a gold standard—gel electrophoresis [17]. However, significant discrepancies remain even between electrophoretic analyses, probably due to investigation of populations with different AS severity [13]. We used, for the first time, the novel latex-based immunoturbidimetric test to measure vWF activity [15]. The vWF:Ac assay seems to be less affected by high bilirubin, free hemoglobin, lipidemia or genetic polymorphism than vWF:RCo and, therefore, allows for more reliable screening of avWS with loss of HMW multimers—especially in the setting of heart valve disease and mechanical circulatory support [18, 19].

We observed similar incidence rates of vWF abnormalities to previous studies utilizing functional vWF assays (vWF:RCo and vWF:CB) in both TAVI and SAVR groups [10, 11]. Comparable values of preprocedural vWF parameters in transcatheter and surgical cohorts suggest that loss of HMW multimers is rather independent from baseline clinical characteristic (low risk vs high risk). Along with a clear correlation between vWF:Ac and transvalvular gradients, evidence support the idea of vWF proteolysis induced by shear-stress, legitimizing recently proposed by Van Belle et al. the series circuit model for quantification of heart valve disease severity based on HMW multimers in the peripheral blood [13].

In 1988, Weinstein et al. described correction of vWF abnormalities after SAVR, confirmed further by Vincentelli et al. [7, 20]. In the following years, several other studies showed normalization of vWF defects after SAVR as well as TAVI [7, 21–28]. Accordingly, in majority of our patients HMW multimers recovered on the first postprocedural day [7, 21–23]. However, to the best of our knowledge, we report for the first-time comparative data on efficacy of both methods. While Bander et al. showed superior effect of SAVR over balloon aortic valvuloplasty (BAV) on the vWF function in stenotic patients, we found SAVR and TAVI to be equivalent treatment options [10]. Moreover, improvement of vWF parameters was observed in all patients, contrarily to Caspar et al., who evidenced positive effect of procedure mainly in cohort with pre-existing vWF abnormalities [11]. The dynamic of improvement in patients with vWF abnormalities differed, however, between transcatheter and surgical patients. In TAVI group increment of vWF parameters was achieved more rapidly on the first postoperative day, whereas surgical group noted significant improvement also on the third day. Variability in vWF increase indicate potential differences between interventions, but also disparities regarding individual vulnerability to multimer degradation. Both need to be further explored for better risk stratification.

Interestingly, literature concerning the association of vWF abnormalities with perioperative bleeding is lacking the evidence. Dysfunctions of vWF remain often undiagnosed, thus their impact on bleeding complications is probably underestimated [13]. Nevertheless, we found TAVI to be unaffected by vWF abnormalities as opposed to SAVR. Majority of bleeding complications in transcatheter cohorts is access-site related and caused rather by anatomical than hemostatic elements. On the other hand, wound surfaces and drainage sites of SAVR, when subjected to dysfunctional vWF, may trigger increased bleeding. Indeed, none of previous studies on vWF in TAVI found relationship between vWF abnormalities and excessive periprocedural bleeding [29]. Data on SAVR is inconsistent, however factors like type of the sternotomy or duration of cardiopulmonary bypass (CBP) might influence the results.

There are several limitations to our work. Firstly, study is inherent to the limitations of any non-randomized small series. Secondly, electrophoretic analysis of vWF multimers to confirm loss of HMW was not performed. Thirdly, mid- and long-term evaluation of primary disorder parameters were not included. Finally, we were unable to measure impact of postprocedural aortic regurgitation on vWF parameters due to low incidence of moderate and severe cases.

## Conclusions

The present findings indicate that both TAVI and SAVR are effective treatment for vWF abnormalities, but vWF abnormalities are predictive of MLTB only in surgical patients.

## Electronic supplementary material

Below is the link to the electronic supplementary material.

Supplementary material 1 (DOCX 6763 kb)

Supplementary material 2 (TIFF 3674 kb)

Supplementary material 3 (TIFF 3378 kb)
