# Supplementary material for: Pre-procedural abnormal function of von Willebrand Factor is predictive of bleeding after surgical but not transcatheter aortic valve replacement
Source: J Thromb Thrombolysis. 2019 Jul 29;48(4):610–8. doi: 10.1007/s11239-019-01917-7 (PMC6800844; doi:10.1007/s11239-019-01917-7)

**Postprocedural evolution of vWF parameters in relation to the size of bioprosthesis:**

**TAVI.** There were no disparities between different transcatheter heart valves (THV) in vWF:Ac/Ag ratio values achieved at day 3 (0.95 ± 0.08 [for 23 mm] vs 1.03 ± 0.06 [for 25 mm] vs 1.06 ± 0.10 [for 26 mm] vs 1.02 [for 27 mm] vs 0.99 ± 0.13 [for 29 mm]; p=0.160; Supplementary Fig 3a). Similarly, relative increase of vWF:Ac/Ag ratio at day 3 in comparison to baseline did not differ between sizes of THVs (37 ± 68% [for 23 mm] vs 19 ± 12% [for 25 mm] vs 29 ± 27% [for 26 mm] vs 22% [for 27 mm] vs 20 ± 25% [for 29 mm]; p=0.900; Supplementary Fig 3b).


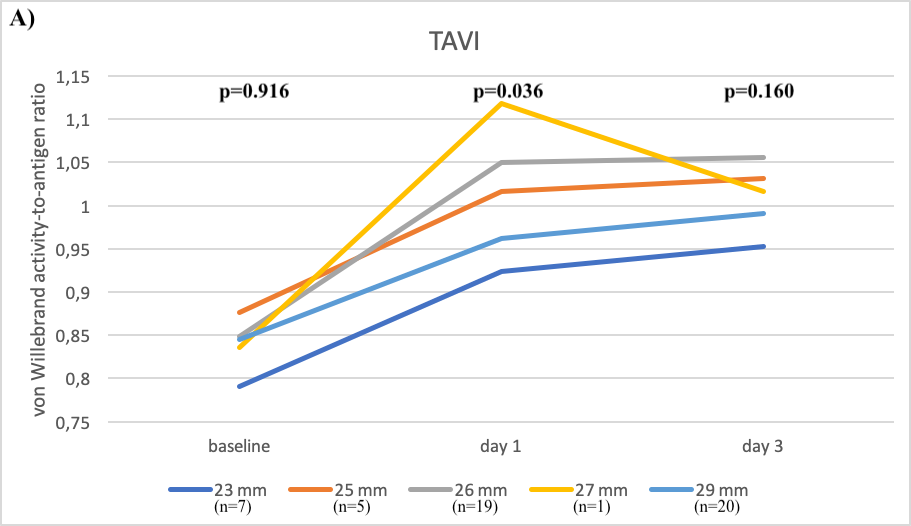


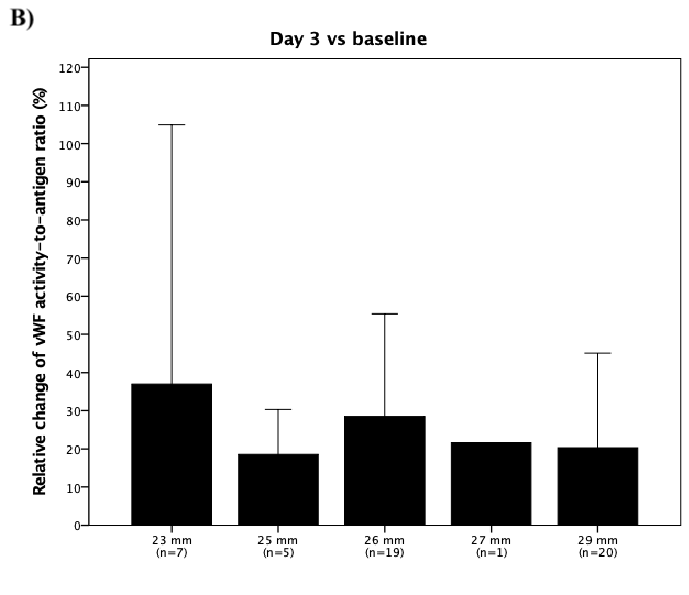


**SAVR.** No disparities were found between different surgical heart valves in vWF:Ac/Ag ratio values achieved at day 3 (0.92 [for 17 mm] vs 0.92 [for 19 mm] vs 1.03 ± 0.16 [for 21 mm] vs 1.05 ± 0.12 [for 23 mm] vs 1.03 ± 0.10 [for 25 mm] vs 1.09 ± 0.09 [for 27 mm] vs 0.93 [for 29 mm]; p=0.433; Supplementary Fig 4a). Correspondingly, relative increase of vWF:Ac/Ag ratio at day 3 in comparison to baseline did not differ between sizes of surgical bioprostheses (24% [for 17 mm] vs 2% [for 19 mm] vs 32 ± 27% [for 21 mm] vs 21 ± 24% [for 23 mm] vs 25 ± 24% [for 25 mm] vs 25 ± 1% [for 27 mm] vs 2% [for 29 mm]; p=0.552; Supplementary Fig 4b).


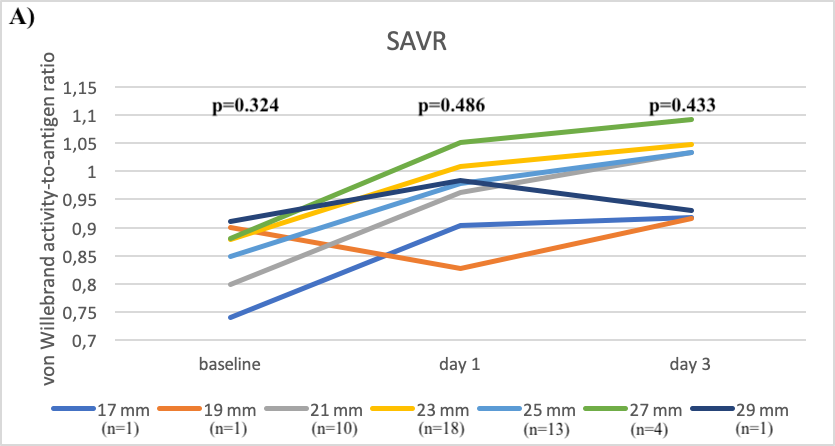


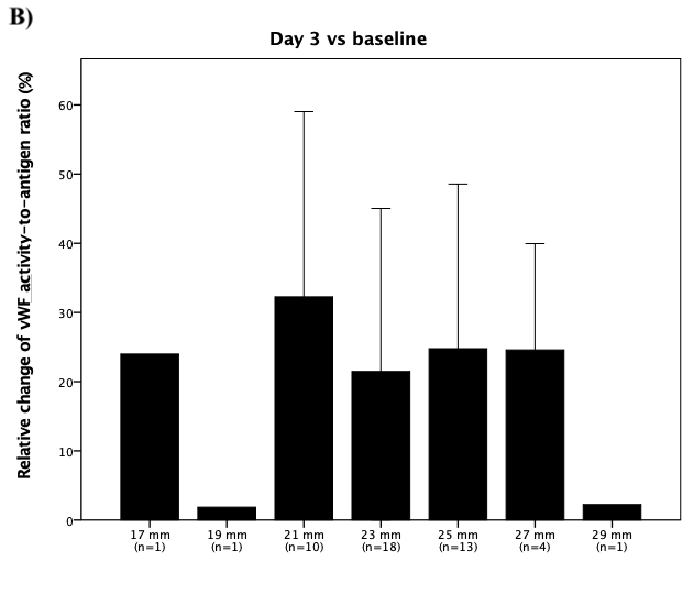

Supplement: Supplementary file 1 — Supplementary material 1 (DOCX 6763 kb) [file 11239_2019_1917_MOESM1_ESM.docx]
