# Supplementary figures and images for: Pre-procedural abnormal function of von Willebrand Factor is predictive of bleeding after surgical but not transcatheter aortic valve replacement
Source: J Thromb Thrombolysis. 2019 Jul 29;48(4):610–8. doi: 10.1007/s11239-019-01917-7 (PMC6800844; doi:10.1007/s11239-019-01917-7)

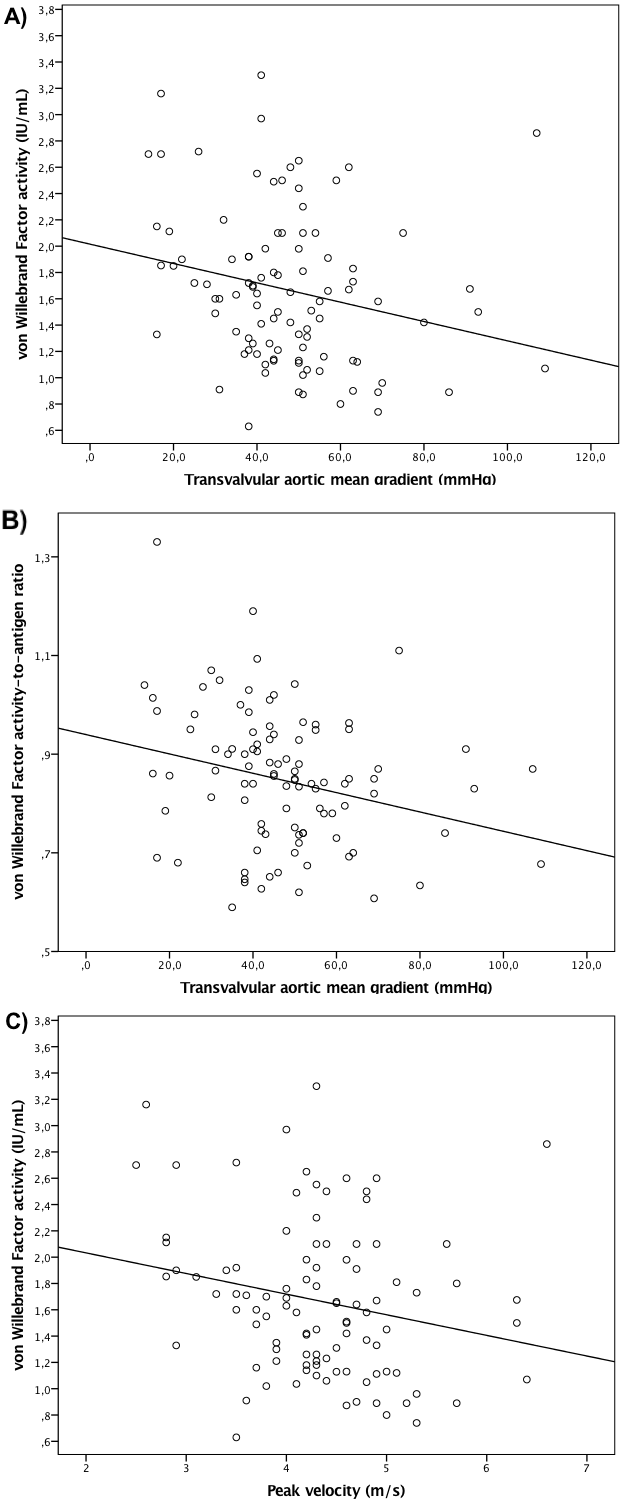

Supplement: Supplementary file 2 — Supplementary material 2 (TIFF 3674 kb) [file 11239_2019_1917_MOESM2_ESM.tif]

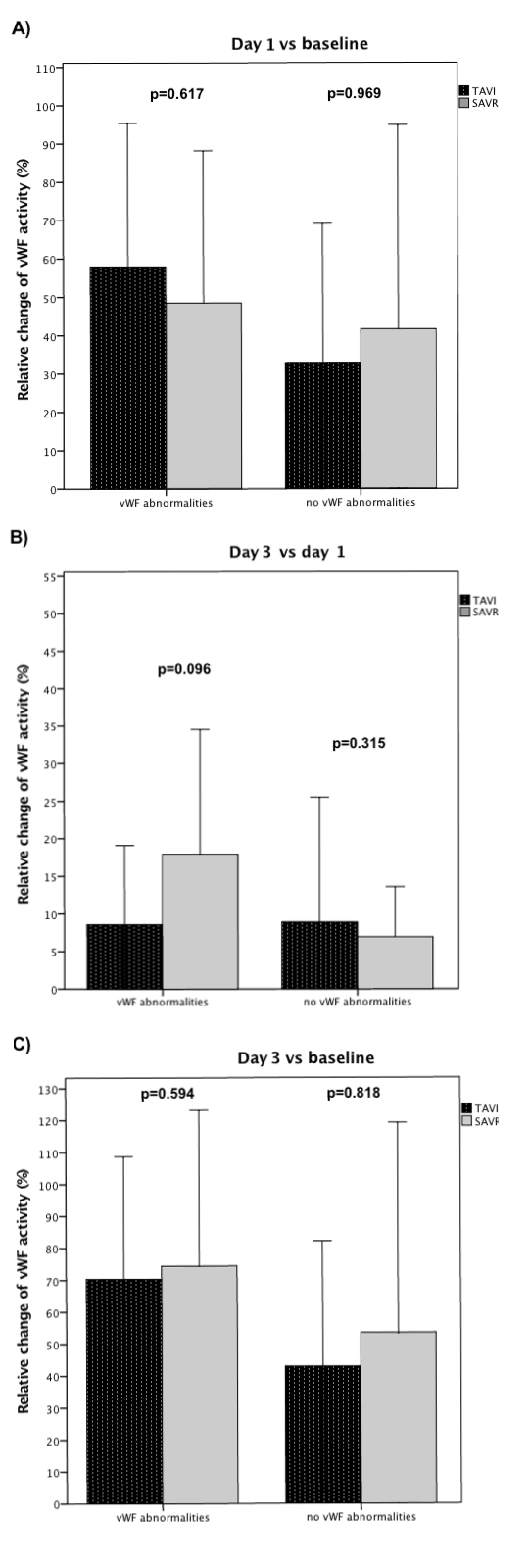

Supplement: Supplementary file 3 — Supplementary material 3 (TIFF 3378 kb) [file 11239_2019_1917_MOESM3_ESM.tif]
